# OLDER WOMEN WITH HIV: STRATEGIES FOR HEALTHY AGING

**DOI:** 10.1093/geroni/igz038.2811

**Published:** 2019-11-08

**Authors:** Mark Brennan-Ing, Liz Seidel, Rebecca Erenrich, Stephen E Karpiak, Ryann N Freeman, Megan Johnson Shen, Chelsie O Burchett, Eugenia L Siegler

**Affiliations:** 1 Brookdale Center for Healthy Aging, Hunter College, The City University of New York, New York, New York, United States; 2 ACRIA Centers at GMHC, new York, New York, United States; 3 ACRIA Centers at GMHC, San Francisco, California, United States; 4 Hennepin County Human Services & Public Health Department, Minneapolis, Minnesota, United States; 5 Weill Cornell Medical Center, new York, New York, United States

## Abstract

Women over 50 represent one-quarter of U.S. older adults with HIV, but we know little about how these women face the challenges of aging. As part of a larger study, we conducted two focus groups with racially diverse older women with HIV in New York City (n = 9) and Oakland, CA (n=11). Discussions concerned strategies for healthy aging. Transcripts were analyzed using inductive thematic methods. Women in both groups shared that they had close connections to family members and the importance of family support for motivating them to take care of their health. Some took proactive steps to stay healthy through nutrition and exercise, although the cost of gym memberships was a barrier. Many were lonely and needed socialization opportunities. Some suggested exercise classes could help to both maintain health and bolster social connections. Implications of these findings for developing programs for older women with HIV will be discussed.

